# Advanced Electrospun Composites Based on Polycaprolactone Fibers Loaded with Micronized Tungsten Powders for Radiation Shielding

**DOI:** 10.3390/polym16182590

**Published:** 2024-09-13

**Authors:** Chiara Giuliani, Ilaria De Stefano, Mariateresa Mancuso, Noemi Fiaschini, Luis Alexander Hein, Daniele Mirabile Gattia, Elisa Scatena, Eleonora Zenobi, Costantino Del Gaudio, Federica Galante, Giuseppe Felici, Antonio Rinaldi

**Affiliations:** 1 TERIN-DEC-ACEL Laboratory, ENEA—Italian National Agency for New Technologies, Energy and Sustainable Economic Development, Via Anguillarese 301, 00123 Rome, Italy; 2 Division of Biotechnologies (SSPT-BIOTEC), ENEA—Italian National Agency for New Technologies, Energy and Sustainable Economic Development, Via Anguillarese 301, 00123 Rome, Italy; ilaria.destefano@enea.it (I.D.S.); mariateresa.mancuso@enea.it (M.M.); 3 NANOFABER S.r.l., 00123 Rome, Italy; noemi.fiaschini@nanofaber.com (N.F.);; 4 SSPT-TIMAF-MADD Laboratory, ENEA—Italian National Agency for New Technologies, Energy and Sustainable Economic Development, Via Anguillarese 301, 00123 Rome, Italy; daniele.mirabile@enea.it; 5 E. Amaldi Foundation, Via del Politecnico snc, 00133 Rome, Italy; elisa.scatena@fondazioneamaldi.it (E.S.); eleonora.zenobi@fondazioneamaldi.it (E.Z.); 6 Italian Space Agency, Via del Politecnico snc, 00133 Rome, Italy; costantino.delgaudio@asi.it; 7 S.I.T. Sordina IORT Technology S.p.A., 04011 Aprilia, Italy; federicagalante95@gmail.com (F.G.); giuseppe.felici@sordina.com (G.F.)

**Keywords:** shielding, polycaprolactone (PCL), tungsten powder, polymer composites, electrospinning, radiation protection, aerospace, healthcare industry

## Abstract

Exposure to high levels of radiation can cause acute, long-term health effects, such as acute radiation syndrome, cancer, and cardiovascular disease. This is an important occupational hazard in different fields, such as the aerospace and healthcare industry, as well as a crucial burden to overcome to boost space applications and exploration. Protective bulky equipment made of heavy metals is not suitable for many advanced purporses, such as mobile devices, wearable shields, and manned spacecrafts. In the latter case, the in-space manufacturing of protective shields is highly desirable and remains an unmet need. Composites made of polymers and high atomic number fillers are potential means for radiation protection due to their low weight, good flexibility, and good processability. In the present work, we developed electrospun composites based on polycaprolactone (polymer matrix) and tungsten powder for application as shielding materials. Electrospinning is a versatile technology that is easily scalable at an industrial level and allows obtaining very lightweight, flexible sheet materials for wearables. By controlling tungsten powder size, we engineered homogeneous, stable and processable suspensions to fabricate radiation composite shielding sheets. The shielding capability was assessed by an in vivo model on prototype composite sheets containing 80 w% of W filler in a polycaprolactone (PCL) fibrous matrix by means of irradiation tests (X-rays) on mice. The obtained results are promising; as expected, the shielding effectivity of the developed composite material increases with the thickness/number of stacked layers. It is worth noting that a thin barrier consisting of 24 layers of the innovative shielding material reduces the extent of apoptosis by 1.5 times compared to the non-shielded mice.

## 1. Introduction

High-energy radiation such as X-ray or gamma ray electromagnetic radiation are often employed or encountered in a wide range of fields, including aerospace and healthcare. Unwanted and/or prolonged exposure to these radiations may be hazardous to health [1,2].

Cosmic radiation, for example, imposes important safety concerns for space exploration missions. Numerous studies have examined the risks associated with exposure to both galactic cosmic rays (GCR) and solar particle events (SPE) [3]. Extended or high-dose radiation exposure can lead to carcinogenesis, cellular mutations, cardiovascular issues, cataracts, and other acute radiation syndromes [1]. Conversely, radiotherapy is frequently employed in medical applications to treat cancer patients. High-energy ionizing radiations (up to tens of MeV) are routinely used to manage tumor growth as part of targeted cancer therapies. The tissues near the treated region are often exposed to penetrative X-rays, which leads to harmful side effects. In addition, X-rays with energies in the keV range are often employed in interventional procedures and in diagnostic radiology such as computed tomography (CT) examination [1,4,5].

Therefore, with the aim to prevent occupational hazards from these kinds of exposures, in the last decade, there has been an increasing demand and research interest in the development of efficient, lightweight, low cost and flexible shielding materials for protection against radiation in the aerospace and medical field [6]. The shielding effectiveness of a given material largely depends on the type of radiation and the range of energies associated with it. Lead (Z = 82) and other high Z materials are known to effectively absorb high-energy radiation and have been commonly used as shielding materials for radiation such as X-rays. [2,4,7,8] Unfortunately, protective equipment made of metals is heavy and bulky, which are often unwanted features for many applications, such as for example for mobile devices, wearable shields, and manned spacecrafts, where there is an unmet need for radioprotective materials that are lightweight and flexible [1].

In this context, polymer composites have become attractive candidates for developing shielding materials for different types of radiation, including high-energy radiation. In particular, polymer composites, prepared by dispersion of metallic or ceramic micro- or nanoparticles (fillers) in the base polymeric matrix, have been found potentially interesting as protective shields [1,2,9,10,11]. The given combination of the filler-matrix pair determines the shielding effect. The role of the filler(s) is to attenuate effectively the radiation, while the polymer offers the mechanical backbone, ensuring good geometric conformability. Polymer is generally less dense than its metal or ceramic counterpart and can be easily processed. Because of the large surface-area-to-volume ratio, micro and nanoparticles have been reported to show enhanced ability to absorb photons [8]. Lead and its compounds have traditionally been used as attenuation fillers due to their low cost, high density, and effectiveness in reducing ionizing radiation [2]. However, lead is highly toxic, and prolonged exposure can cause serious health problems such as neurological disorders, kidney failure, and a decrease in hemoglobin and red blood cells. As a result, it is crucial to limit the use of lead and explore alternative metals and synthetic materials that are less toxic [12,13]. Eco-friendly shielding materials that can replace lead include tungsten (W), bismuth oxide (Bi_2_O_3_), barium sulfate (BaSO_4_), and boron [14,15,16]. Among these materials, W has an atomic number of 74, which is similar to that of lead (82), offers high density (19.25 g/cm^3^), is characterized by low toxicity among the high-Z elements [17], exhibiting a comparable shielding effect [2,18]. For instance, Selyutin et al. studied ultra-high molecular weight polyethylene (UHMWPE) doped with tungsten powder, demonstrating that UHMWPE is resistant to ionizing radiation [19]. The radiation shielding properties of tungsten and epoxy composite were also investigated by Le Chang et al. [20]. Moreover, Soylu et al.’s research showed that the composites’ radiation shielding efficiency based on tungsten carbide and ethylene vinyl acetate polymer is similar to lead [21].

In this work, innovative, flexible hybrid composite sheets made of a polycaprolactone (PCL) fibrous matrix containing W powder were prepared via electrospinning. PCL is a biodegradable polymer [22] commonly used for various applications due to its ease of processing [23]. To the best of our knowledge, PCL-based shielding composites are reported in literature only for EMI [24,25] and microwave shielding [26,27]. Electrospinning is a relatively simple, low-cost, and versatile technology that allows obtaining innovative micro- and nano-structured non-woven materials for application in several fields [28]. The developed electrospun composites were structurally and physically characterized, and their ability to shield high-energy radiation (X-rays) was investigated through in vivo tests.

## 2. Materials and Methods

Commercially available tungsten, W, powder 99+ (fine powder < 20 μm, purity 99%, Sigma Aldrich, St. Louis, MI, USA), polycaprolactone (PCL) polymer (6800D, 80 kDa, Perstorp, Warrington, UK), chloroform (VWR) and dimethylformamide (DMF), (VWR) were used for the preparation of PCL/W based suspensions. Ethanol (Carlo Erba) was used as a penetrating liquid to determine the porosity of the prepared electrospun materials. A commercial polylactid acid (PLA) filament (FILOALFA, Turin, Italy; 1.75 mm diameter) was used for the 3D printing process of the cylindrical samples.

### 2.1. Optimization of W Powder

W powder was selected as a filler for the preparation of a PCL-based shielding sheet. Commercial W powder was used as the starting material for the filler. Ball milling refined the size of commercial W particles to obtain a stable suspension processable via an electrospinning technique. The ball milling process was carried out in a SPEX 8000 mixer/mill. The W powder was ball-milled in stainless steel vials. The upper cup was allowed to seal the vial by the mean of a viton o-ring. To avoid contamination, the jars were loaded with powder and spheres in a glove box. The powders were ball milled in a slight overpressure of gaseous Argon. After different tests, an optimized powder, in terms of morphological and microstructural properties, was obtained after 26 h of ball milling.

### 2.2. Electrospinning of PCL/W-Based Shielding Materials

PCL/W sheets were produced via electrospinning using needle-technology electrospinning equipment (Fluidnatek LE100, Bioinicia, Paterna, Spain) outfitted with a flat collector and two-axis emitter motion. The polymer solution was prepared by dissolving the 12% *w*/*w* of PCL in the chloroform/dimethylformamide 65/35 solvent mixture at room temperature. Different amounts of the optimized W powder (10–80 w% with respect to the PCL polymer) were added to the PCL solution to optimize the properties of the resulting microfibrous sheets.

The electrospinning process and the properties of the resulting electrospun materials are highly dependent on process parameters and ambient conditions [29]. The process parameters optimized for the preparation of the PCL-based sheets are summarized in the following table (Table 1).

The relative humidity and the temperature in the process chamber were kept constant at average values of 47% (with a mean square error (MSE) of ±2%) and 25 °C (with an MSE of ±0.2 °C), respectively, throughout the entire process.

### 2.3. Scanning Electron Microscopy (SEM) and Energy Dispersive X-ray Spectroscopy (EDS)

The morphological properties of the PCL/W electrospun material were analyzed using a field emission gun scanning electron microscope (FEG-SEM) model Leo 1530 (ZEISS, Oberkochen, Germany), operating at a low voltage (2 kV) to prevent charging effects and overheating damage to the dielectric polymer. The membrane thickness was assessed by examining the sample’s cross-section under the SEM. Additionally, energy-dispersive X-ray spectroscopy (EDS) measurements were performed with an X-MAX detector (AZ-TEC, Oxford, UK) to verify the presence of W in the PCL samples.

### 2.4. Porosity and Permeability Determination

The porosity of the membranes was assessed using the liquid displacement method [30,31,32]. Ethanol was chosen as the penetrating liquid due to its ability to permeate the porous membrane and its low density (≈0.790 g/mL). Percent porosity (ε%) was calculated using Equation (1):

(1) $$\varepsilon_{\%}=\frac{\left(m_{3}-m_{4}-m_{1}\right)}{m_{2}-m_{4}}$$

Weights m_1_, m_2_, m_3_, and m_4_ were measured on an ORMA scale (BCA120, Milan, Italy) at 20 °C. For each specimen, m_1_ represents the weight of the “dry” sample; m_2_ is the weight of a “control” graduated bottle filled with ethanol up to a specified volume; m_3_ is the weight of the same bottle after the specimen has been added and immersed in ethanol, with excess ethanol carefully removed to restore the control volume and m_4_ is the weight of the bottle after removing the wet sample and leaving residual ethanol.

Air permeability of the electrospun membranes was evaluated using a Gurley densometer (model 4320, Troy, NY, USA). During the test, the time (t) in seconds for a volume (V) of 100 cm^3^ of air to pass through a surface area (A) of 1.61 cm^2^ was recorded. The percent permeability value (P%) in “cm/s” for each sample was determined using the following Equation (2) [33].

(2) $$P\%=\frac{V}{A\cdot t}\;\cdot 100$$

### 2.5. Mechanical Tests

The tensile properties of the PCL/W-based shielding materials were evaluated by a tensile loading frame (Dynamic Mechanical Analyzer 850, TA Instruments, New Castle, DE, USA) equipped with a 20 N load cell. Rectangular strips were cut and mounted in the loading frame, with a free gauge length of 10–15 mm and a uniform width (w) of 4 mm. The sample thickness (s) was measured by SEM imaging of the cross-section. Load vs. cross-head displacement data was recorded during tensile tests (movable cross-head moving at 0.2 mm/min) at room temperature. The engineering stress (σ, MPa) vs. strain (ε, %) curves were calculated by dividing, respectively, the applied load by the apparent cross-sectional area (A_0_ = w × s) and the cross-head displacement by 10, corrected for elongation amount, needed to fully stretch the strip. The Young modulus (E) was estimated from the slope of the linear fit in the elastic region for each stress–strain curve.

### 2.6. PLA Cylinder Preparation

**Figure 1:**
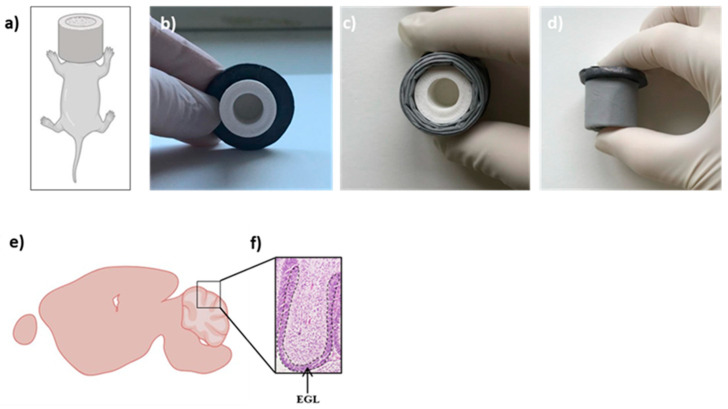
Schematic representation of the experimental setup (**a**), custom-built 3D printed PLA protective device for in vivo test mounted on a 3 mm thick solid lead sheet (**b**); PLA device wrapped up in 12 shielding sheets of PCL/W (**c**); PLA device wrapped up in 24 shielding sheets of PCL/W (**d**); schematic representation of a section of a cerebellum at 2 days of age (**e**); antero-dorsal cardinal lobe of the cerebellum (**f**). The dashed black line externally outlines the EGL. Figure in (**a**,**e**) was obtained by Biorender.

For the in vivo experimental tests, cylindrical PLA supports were 3D printed by means of the fused deposition modeling (FDM) technique (Raise 3D Inc., Irvine, CA, USA). These samples were designed according to the following geometrical specifications: a cylindrical structure of 18 mm outer diameter and 20 mm height, with an inner cavity of 10 mm diameter and 16 mm height for housing the mice head (Figure 1a,b). The PLA filament was extruded through a 0.4 mm diameter nozzle at 205 °C; the bed temperature was set at 60 °C. All samples were fabricated with the same printing parameters.

### 2.7. Irradiation Shielding Tests

#### 2.7.1. Mice

Mice lacking one *Ptch1* allele were bred on CD1 background (*Ptch1^+/−^*) and genotyped according to our previous studies [34]. This animal study was performed according to Directive 2010/63/EU of the European Parliament, approved by the local Ethical Committee for Animal Experiments of the ENEA, and authorized by the Italian Ministry of Health (n°740/2021-PR).

#### 2.7.2. Irradiation Shielding Test

*Ptch1^+/−^* mice at postnatal day 2 (P2) were shielded and irradiated with 1 Gy of X-ray protecting heads with different kinds of shields (Figure 1a). Specifically, one set of mice was irradiated by shielding the head with PLA cylinder (PLA, n = 6; Figure 1b), another set with PLA cylinder wrapped up in 12 sheets of electrospinning PCL/W (N12, n = 6; Figure 1c), and another set with PLA cylinder wrapped up in 24 sheets of electrospinning PCL/W (N24, n = 6, Figure 1d). Another group of animals was whole-body irradiated (WB, n = 6). An additional group of mice was left unirradiated as a control (CN, n = 6). Irradiation was performed using a Gilardoni CHF 320 G X-ray generator (Gilardoni, Mandello del Lario, Italy), operated at 250 kVp, 15 mA, with filters of 2.0 mm of Al and 0.5 mm of Cu. Protection of shielded parts was verified by dosimetry and Monte Carlo simulation.

#### 2.7.3. Apoptosis Quantification in the Cerebellum

To study the short-term cellular responses to radiation exposure, both control and irradiated mice were euthanized by CO_2_ asphyxiation at 4 h post-irradiation. Brains were collected and processed for histological analysis according to standard protocols; 4 µm-thick sections, after Hematoxylin and Eosin staining, were thus observed using light microscopy to quantify the number of apoptotic cells in the cerebellum external granule layer (EGL) (Figure 1e,f) as previously described [35]. The quantitative analysis was performed along the entire antero-dorsal cardinal lobe of the cerebellum and was carried out blindly by two different operators. Samples in which the area of interest could not be analyzed due to poor orientation were excluded from the analysis. The apoptotic rate was calculated as the percentage of pyknotic nuclei relative to the total cell number (Figure 1a–d).

Paraffin brain sections were also processed for the immunohistochemical analysis using an antibody direct against cleaved caspase-3 (Cell Signaling Technology, Danvers, MA, USA; 1:100), an elective marker of apoptosis [36]. Images for quantification were taken using the imaging software NIS-Elements BR 4.00.05 (Nikon Instruments Europe B.V., Amsterdam, The Netherlands).

The colorimetric signal of cleaved caspase 3 was quantified using the HistoQuest software (TissueGnostics, Vienna, VA, Austria) and expressed as the labeled area/total area examined.

#### 2.7.4. Statistics

Analyses were performed using GraphPad Prism 5.0 (GraphPad Software, San Diego, CA, USA). Statistical significance was determined using a two-tailed Student *t*-test. *p*-values < 0.005 were considered statistically significant.

## 3. Results and Discussion

### 3.1. Powder Optimization

In our study, PCL was selected as the polymer matrix for the shielding material due to its good processability, biocompatibility, and thermal stability. The addition of W powder as a filler for the polymer matrix allows us to tune the shielding properties of the material and reduce its weight.

The electrospinning technique offers considerable advantages, such as to easily tune the morphology and the characteristics of the obtained materials and/or to select in wide intervals the polymer matrix, the type and the concentration of the filler. As an electrohydrodynamic process, this technology allows to collect the fibers produced on a counter-electrode without any dispersion of material (unlike wire or powder bed 3D printing technologies, for example). In addition, electrospinning is relatively simple, flexible, and easily scalable at an industrial level and provides very lightweight, flexible and, therefore, wearable TNT materials.

**Figure 2:**
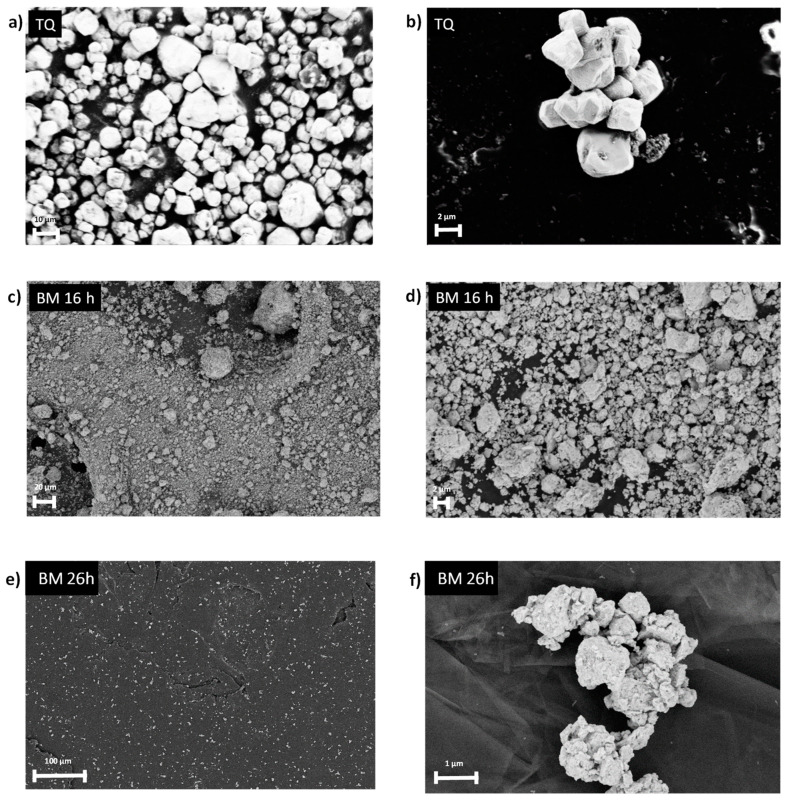
The SEM image of the commercial W powder is as follows: before (**a**,**b**), after 16 h (**c**,**d**) and 26 h (**e**,**f**) of ball milling.

**Figure 3:**
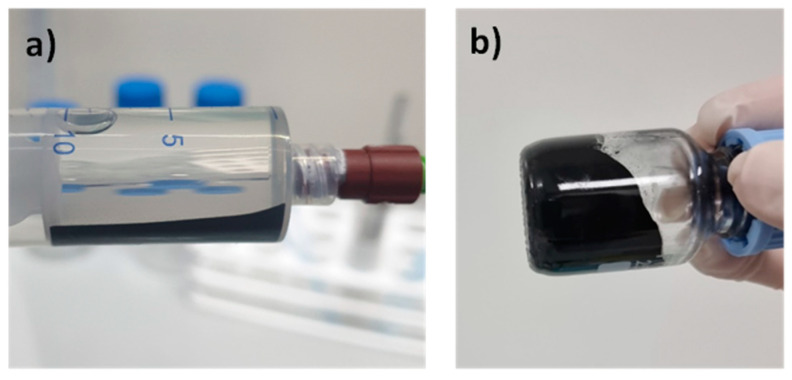
Unstable suspension prepared using PCL and the commercial W powder (**a**), and stable suspension based on PCL and the optimized W powder (**b**).

It is worth noting that the particle size of the powder plays a key role in the physical stability of the polymer/W suspension. Therefore, the control of W particle size is essential in achieving a homogeneous, stable, and processable suspension. The selected commercial W powder, with a maximum particle size of 10 µm (Figure 2a,b), was unsuitable for preparing stable suspensions. In fact, W particles remained in suspension with the polymer only with the aid of magnetic stirring, while they settled in a few minutes after stopping the external agitation (Figure 3). In this case, the suspension is not suitable to be processed by electrospinning, requiring continuous stirring in situ to avoid powder settling.

Ball milling is a powerful method to refine powder particle dimensions, particularly in refractory metals like W and oxides. Our goal was thus to optimize the W particle size by ball milling to obtain a stable PCL/W suspension suitable for deposition by electrospinning.

After 26 h of milling, the SEM observation revealed that the diameter of the final particles was more homogeneous and significantly reduced to values equal or less than 1 µm (Figure 2e,f). The obtained powder was mixed with PCL to prepare a stable PCL/W suspension. As shown in Figure 3, the prepared suspension was homogenous and stable for several hours, even without stirring; thus, it was suitable to be processed by the electrospinning technique.

### 3.2. Preparation and Microstructural Characterization of PCL/W-Based Shielding Materials

**Figure 4:**
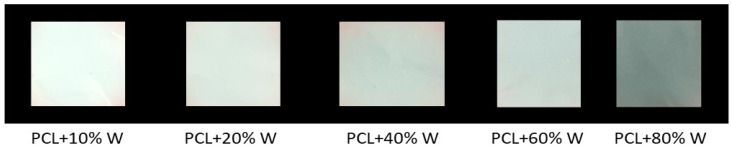
Micro-fibrous PCL sheets characterized by different W content (w% with respect to the PCL polymer).

Five batches of PCL/W sheets were prepared via electrospinning and characterized. To tune the electrospun material’s final properties, different amounts of optimized W powder (in a 10–80 w% range with respect to the PCL polymer) were successfully added to the PCL solution. For the preparation of the PCL/W sheet here reported, the optimized W powder was ball-milled for 26 h. The prepared suspensions were processed via electrospinning to obtain the micro-fibrous sheets shown in Figure 4.

The increase of the W content is apparent at a glance by visual inspection; the darker the samples, the higher the percentage of the metallic filler.

**Figure 5:**
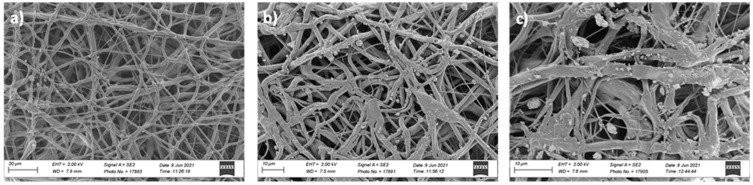
Microfibrous PCL/W sheets characterized as 10 w% (**a**), 40 w% (**b**), and 60 w% (**c**) of W with respect to the PCL polymer.

**Figure 6:**
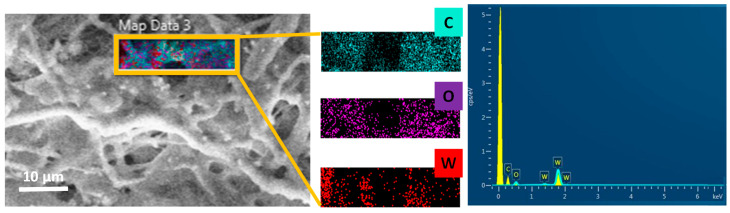
EDS analysis of the PCL/W electrospun sheets.

The morphology of the PCL/W sheets was investigated using SEM. Some representative micrographs of the obtained sheets are reported as an example in Figure 5. SEM images show that the electrospun samples are basically composed of a dense net of randomly oriented fibers. In addition, SEM analysis revealed the presence of the W particles in the PCL micro-fibrous network. It is evident from the images in Figure 5 how the amount of W particles/aggregates increases with increasing the filler percentage, thus confirming the visual appearance of the samples. EDS analysis at high resolution also confirmed the presence of W in the sheets (Figure 6).

In addition, the presence and the amount of W filler affect the mean fiber diameter (Table 2). The addition of W powder to PCL increases the diameter of fibers with respect to those of pure polymer. Increasing the filler content by up to 80% means that the mean fiber diameter and standard deviation grow significantly. This leads to an overall structure that is less homogeneous and dense in fibers.

### 3.3. Porosity and Air Permeability Analysis

A more detailed characterization was carried out on the most promising sample, the one containing the higher percentage of the W filler (PCL/W80); the neat PCL mat was used as a reference. In particular, the porosity and the air permeability of the electrospun PCL/W80 were evaluated, and the average values obtained from three measurements of each membrane are reported in Table 2.

Regarding the analysis of the overall porosity, it was carried out using the volume displacement method [31,32]. The electrospun PCL’s porosity was 61%, a value consistent with the values reported in the literature (typically between 60% and 90%) [37]. The porosity describes the volume of voids inside a given volume of a fiber mat. The total volume of voids depends both on the volume and on the number of pores. The porosity of the pure PCL/W80 electrospun sample was found to be lower than that of the neat PCL. This is consistent with the results of the mean fiber diameter analysis. The fiber size is the key parameter that determines the pore size of an electrospun fiber web. An increase in the nanofiber diameter leads to a lower number of pores with larger dimensions [38]. In the specific case of the sole PCL sample, a dense net of thin fibers results in a dense structure of small pores. On the contrary, the PCL/W80 sample is thinner than the PCL and is formed by a less dense net of coarse fibers than the PCL sheets. The result is an overall structure with larger pores than PCL and fewer pores.

Regarding air permeability, the web with low porosity is expected to exhibit poorer transport properties. It must be mentioned that the net morphology significantly influences the permeability of air through it. Pore characteristics, such as dimension, directly influence the air permeability of electrospun webs. The results show that the PCL/W80 sample exhibits higher air permeability than PCL. This can be interpreted as the result of the fact that more resistance in airflow through the smaller pores has occurred.

### 3.4. Mechanical Characterization

To evaluate the influence of the incorporation of the W filler on mechanical performance of the PCL-based electrospun sheets, tensile tests were performed. Thickness analysis (by SEM cross-sectional analysis) allowed also to evaluate and compare the mechanical properties of the different samples. The obtained results are shown in Table 3.

Young’s modulus was lower in the composite sheet than in the pure one, indicating a stiffness decrease due to fillers. Ramier et al. have noticed that hydroxyapatite nanoparticle incorporation within the electrospun poly(3-hydroxybutyrate) fibers (PHB/nHA (blend)) significantly improved the mechanical properties of the fiber mat [38]. Similarly, numerous research articles report an enhancement of mechanical properties, including elastic modulus and tensile strength, after the addition of various carbon fillers [39,40]. On the other hand, in the presence of a high filler content, a reduction in crystallinity may occur. In fact, the formation of filler aggregates would restrain polymer chains’ mobility and hinder their ordered arrangement, adversely affecting the crystallization process and thus hindering crystallization [41,42]. Accordingly, we can assume that the lower Young’s modulus of the PCL/W80 composite correlates with a reduction in crystallinity due to filler aggregates, as observed in the SEM images (Figure 6).

However, it is worth noting that, in the presence of a W content up to 80 w% with respect to the PCL, the electrospun sheet maintains the pure polymer’s ductility and plastic behavior.

### 3.5. Assessment of the Shielding Effect of the Optimized Formulation (PCL/W80): In Vivo Test

The EGL is a region of the cerebellum that undergoes active proliferation and differentiation during early postnatal development. Because of its high proliferative activity, it is used as a model system to study the effects of radiation on developing neural tissue [36,43]. In fact, by assessing the extent of radiation-induced damage, including apoptosis, in the EGL, it is possible to establish a dose-effect relationship, which allows for determining how the severity of damage changes with increasing doses of radiation [35]. Studying the dose-effect relationship in the EGL can thus provide insights into the mechanisms underlying radiation-induced damage to developing neural tissue and potential strategies for mitigating or preventing such damage.

**Figure 7:**
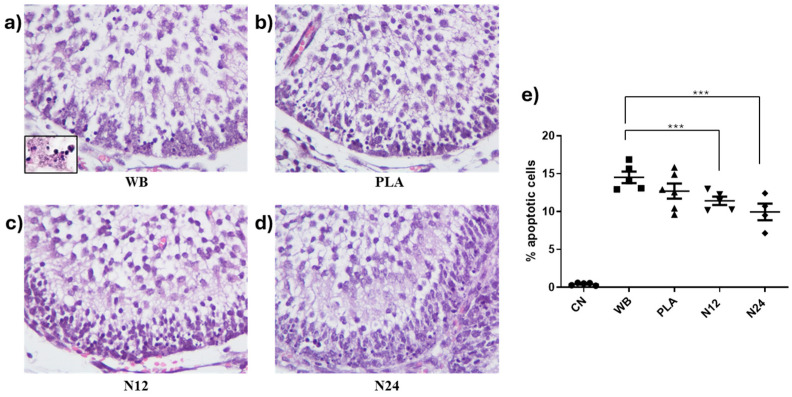
The apoptotic rate in EGL of irradiated mice under different experimental conditions. (**a**–**d**) Representative images of the EGL in the antero-dorsal cardinal lobe region of the cerebellum at postnatal day 2 (P2); Hematoxylin & Eosin staining; 40× magnification. The inset in (**a**) shows pyknotic nuclei at higher magnification (100×) indicative of apoptosis in WB irradiated mice; (**e**) a graphical representation of the percentage of apoptotic cells in the different experimental groups. *** *p* < 0.0001.

In our experimental setup, we evaluated the apoptotic rate induced by a single dose of 1 Gy of X-rays to determine the effectiveness of PCL/W80 sheets in attenuating DNA damage. As expected, rare apoptotic cells were detected in the cerebella of non-irradiated animals (0.4302% ± 0.17). On the contrary, in WB-irradiated mice, the average percentage of apoptosis was 14.5% ± 0.76, a highly significant value compared to the unirradiated group (*p* < 0.0001; Figure 7a–e). In the EGL of PLA-shielded mice, the percentage of apoptosis did not differ significantly compared to the WB group, suggesting that PLA does not possess shielding capacity (12.70 ± 0.98%, *p* = 0.1941; Figure 7b–e). In both the N12 and N24 configurations, a significant reduction in cell damage is observed compared to the WB group. Notably, the N24 setup reduces the extent of apoptosis to 9.94% ± 1.11 by 1.5 times compared to the irradiated group and by 1.28 times compared to the PLA group. Although this latter value does not reach statistical significance, it suggests that the dose reduction provided by the PCL/W80 shielding is proportional to the thickness of the layer obtained from the sheets.

**Figure 8:**
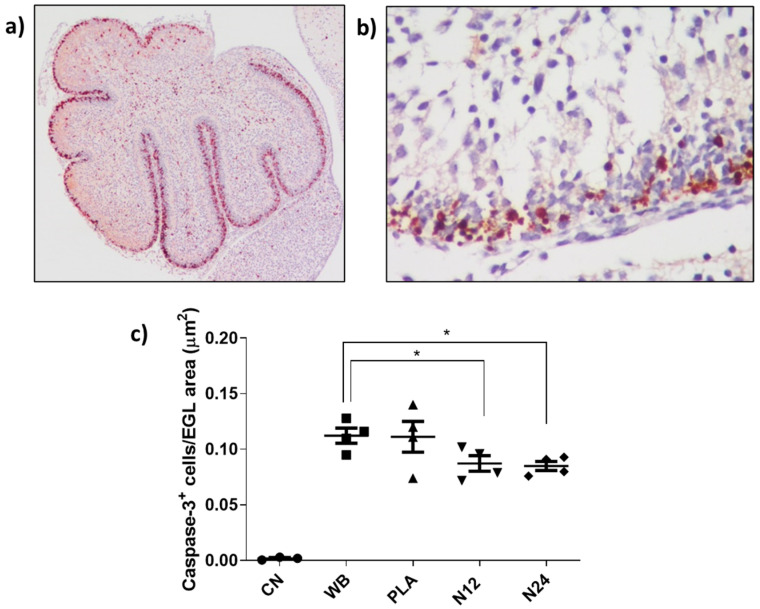
(**a**) Cerebellum at P2 labeled with activated caspase 3; the EGL, where the cells undergoing apoptosis reside, is colored red (2× magnification). (**b**) Detail of the EGL at higher magnification (40×). (**c**) Graphical representation of the quantification of the signal related to the antero-dorsal cardinal lobe region. * *p* < 0.05.

To validate the data obtained based on morphological criteria, we evaluated apoptosis by adopting an immunohistochemical approach using the cleaved caspase 3 antibody as an apoptotic marker. As shown in Figure 8a,b, apoptosis detected by the antibody is exclusively present in the EGL of the mouse cerebellum. Quantifying the ratio of labeled EGL to the total examined area for each experimental group, results confirmed the effectiveness of PCL/W 80% sheets in attenuating the apoptosis rate compared to that induced in the WB group (*p* < 0.0439 for N12; *p* < 0.0145 for N24) (Figure 8c). Concordantly, the region of interest in the control group showed negative results for caspase 3.

## 4. Conclusions

In the present study, we proposed the development of shielding materials consisting of an electrospun PCL matrix functionalized with W powder (filler). Particle size plays a key role in the obtainment of homogeneous and stable suspensions processable via electrospinning; the particle dimension of a commercial W powder was refined up to 1 µm by 26 h of ball milling. The obtained powder led to homogenous PCL/W suspensions that were stable for several hours and suitable to be processed by electrospinning technique. Different amounts of the optimized filler were added to the polymer suspension, and the resulting batches were electrospun and characterized. The material containing the higher percentage of the W filler, i.e., 80 w% with respect to the PCL polymer (PCL/W 80%), turned out to be very promising and was selected for subsequent investigation. The shielding properties of the electrospun PCL/W 80% sheets were assessed by means of in vivo tests on mice. Specifically, we evaluated the extent of radiation-induced damage, including apoptosis, in the EGL region of mice cerebellum on both whole body and shielded irradiated mice. Different configurations were considered by increasing the number of PCL/W 80% sheets/thickness of the shielding system. The results showed that a significant reduction in cell damage was observed in the shielded groups compared to the whole-body irradiated mice. In particular, the thin barrier consisting of 24 stacked layers of the developed shielding material was found to reduce the extent of apoptosis by 1.5 times compared to the non-shielded mice. As expected, the dose reduction of the shielding is proportional to the thickness of the layer obtained from the sheets. Thus, these findings, combined with a lightweight, flexible, relatively simple, and easily scalable production methodology at an industrial level, are promising for the application of the developed electrospun material for radiation protection in the aerospace and medical fields. The possibility of implementing this electrospinning manufacturing process also in conditions of micro-gravity or absence of gravity is particularly relevant for in-space manufacturing and space applications.

## 5. Patents

Italian patent application (102023000027945, PA104151IT01) filed on 22 December 2023.

## Figures and Tables

**Table 1:** Processing parameters for the electrospinning of PCL PSU/W solutions.

Parameter	Label	Unit	Value
Flow rate	FR	mL/h	6
Voltage at injector	Vi	kV	5
Voltage at collector	Vc	kV	−5
Working distance	d	cm	16
Deposition time	t	min	43
Deposition area	A	cm^2^	144

**Table 2:** Results of the structural characterization of the PCL/W samples containing different amounts of the W filler: the mean fiber diameter (μ), with standard deviation (σ).

Sample	μ (μm)	σ (μm)
PCL	0.60	0.43
PCL/W 10%	0.97	0.37
PCL/W 20%	1.18	0.88
PCL/W 40%	1.17	0.67
PCL/W 60%	1.15	0.89
PCL/W 80%	2.32	1.88

**Table 3:** Results of the characterization of the PCL/W80 and pure PCL samples: the average thickness, the Young’s modulus (E), porosity, ε, and air permeability.

Sample	Average Thickness (μm) *	E (MPa)	ε (%)	Air Permeability (cm/s)
PCL	156	64 ± 2	61	3.99
PCL/W 80%	120	52 ± 2	51	5.67

* ±2%.

## Data Availability

The original contributions presented in the study are included in the article, further inquiries can be directed to the corresponding authors.
